# Linking environmental management accounting to green organisational behaviour: The mediating role of green human resource management

**DOI:** 10.1371/journal.pone.0279568

**Published:** 2022-12-28

**Authors:** Liping Liu, Chunyu Zhang

**Affiliations:** School of Economics and Management, Guangxi Normal University, Guilin, Guangxi, China; Universiti Teknologi Malaysia - Main Campus Skudai: Universiti Teknologi Malaysia, MALAYSIA

## Abstract

The China’s economy is developing rapidly, and it affects the environment on several levels. Therefore, this study examines the effect of environmental management accounting, green human resource management (HRM), on green organisational behaviour (OB). We collected 383 questionnaires completed by human resource managers and accounting managers in the Chinese hospitality industry. We used a covariance-based structural equation model to test the hypotheses in this study. The empirical evidence shows a positive and significant effect between environmental management accounting (monetary environmental management accounting, and physical environmental management accounting) and green HRM (*β* = 0.262, *p* < 0.01, *β* = 0.378, *p*< 0.01). Green HRM mediates the influence of environmental management accounting (monetary environmental management accounting, *β* = 0.059, *p* < 0.01; physical environmental management accounting, *β* = 0.084, *p*< 0.01) on green OB. The analysis confirmed the importance of environmental management accounting (as opposed to green human resource management) in predicting green behaviour and the critical role of green human resource management in connecting environmental management accounting and green OB. Thus, this study extends the literature’s perspective on green OB to environmental management accounting and green HRM.

## 1. Introduction

Ecological and environmental problems have increasingly emerged globally, including global temperature rise, frequent forest fires, and water and soil pollution [1]. The issue of environment and sustainable development has attracted increasing attention among organisations [2]. Faced with severe natural environmental problems, humans must protect the natural environment and show green behaviour. Green behaviour promotes sustainable environmental development, such as ecological protection, resource conservation, and waste conversion. Green organisational behaviour (OB) helps enterprises implement green management and promote the sustainable development of other enterprises. Growing numbers of organisations have begun implementing sustainable development policies and management systems. Stakeholders increased pressure to implement environmental management initiatives to address the intricacy of environmental sustainability [3].

The increasingly fierce demand for environmental protection and the awakening of environmental awareness of the public has gradually become a powerful driving force for green management in organisations. Enterprises’ environmental protection behaviours meet stakeholders’ environmental demands, reduce operating costs, and improve social responsibility [4]. Given the important value of employees’ green behaviour, scholars began discussing which management measures organisations can use to stimulate employees’ green behaviour [5]. In particular, two green management tools—environmental management accounting and green human resource management—have received much attention [6–8].

Environmental management accounting (EMA) manages environmental and economic performance by developing and implementing appropriate environment-related accounting practices [9]. EMA includes monetary environmental management accounting (MEMA) and physical environmental management accounting (PEMA). organisational environmental protection is strongly influenced by EMA, which is why EMA plays a significant role in reducing the environmental burden in the form of energy dependence [8]. Traditional accounting rules do not provide information that can be used to evaluate and monitor the environmental effect of the hospitality industry [10]. In the past, standard operations management approaches were used to evaluate an organisation’s performance based on cost, quality, and profit, with little regard for environmentalism. Previous research has shown EMA mediates the significant positive effect of environmental strategies on the environmental performance of companies [11], the effect of pollution prevention and clean technology strategies on environmental performance [12], and it has a positive association with process innovation [13]. By comparison, EMA is linked to the information requirements of environmental management in organisational decision-making [14]. There is a current concern that environmental problems exist in the context of increased pressure from stakeholders and policy. This study will explore EMA information for the hotel industry’s green behaviour with long and short-term decision-making processes, and the effect of EMA on green OB.

Green human resource management (HRM) refers to a series of positive plans enterprises take to deal with environmental problems. Previous research has shown green human resource management effects green employee behaviour [15], organisational citizenship behaviour for the environment [16]. And green organisational culture, green purchasing, and top management commitment toward greening the workforce are the key antecedents for the exercise of green HRM practices [17]. This study attempts to address a gap in the current literature by enhancing our understanding of such mediations. This study simultaneously examines the effect of MEMA and PEMA on green HRM. This study offers a more fine-grained picture of outcomes relevant to EMA, which separates MEMA and PEMA. Providing such a fine-grained picture is important. It shows that MEMA and PEMA are similarly important for both types of EMA. Knowing the importance of EMA as the meditating variable helps in how it estimates green HRM, in turn, is known to foster green OB [18].

### 1.1 Environmental management accounting and green human resource management

EMA is defined as creating, analyzing, and utilizing financial and non-financial data to enhance corporate environmental and economic performance and accomplish sustainable goals, it covers all aspects of accounting that may be impacted by an organisation’s response to environmental challenges and new eco-efficiency areas [9]. Normally, EMA involves life-cycle costing, full-cost accounting, benefits assessment, and strategic planning for environmental management [19]. EMA promotes public accountability through mandatory reporting on environmental performance [20] and operational performance to improve organisational environmental control and minimize operating costs to enhance profits [21]. EMA includes MEMA and PEMA. MEMA focuses on environmental impact information expressed in monetary units, for example, the costs incurred to treat waste, it provides an important tool to track, trace, and treat costs incurred due to an organisation’s activities relating to the environment [13]. PEMA deals with environmental impact information expressed in physical units (e.g., kilograms) of materials. For example, it is assumed that all physical inputs (energy, water, and materials) eventually become outputs (i.e., physical products, waste, or emissions) and that all physical inputs and outputs should be tracked to ensure that no significant quantities go unaccounted for [13].

EMA helps companies meet environmental responsibility and lead to the economic benefits of improved environmental and economic performance [22]. Moreover, EMA is an approach to disclosing information that assists enterprises in reaching better environmental and financial performance [23]. EMA is also analogous to green HRM, ensuring that companies effectively and efficiently use available green human resources to support environmentalism, green HRM is derived from “environmental management,” which integrates environmental management into enterprise human resource management [24]. It conducts green recruitment, green training, green compensation, green performance, and green employee participation in environmental protection, effectively implements environmental sustainability policies [15], and promotes sustainable development by building human resources with green competitive advantages (such as employees with green consciousness green behaviour). To understand the purpose of green human resources management, some scholars put forward the goals of solving environmental problems and promoting strategic environmental objectives. These goals can help to improve environmental protection mechanisms and aid organisations address regulatory demands through operational control and employee stewardship. Thus, we propose the following hypothesis:

H1a: MEMA will positively effect green HRM.

H1b: PEMA will positively effect green HRM.

### 1.2 Green human resource management and green organisational behaviour

Green HRM is defined as HR policies, philosophies, and practices (recruitment and selection, training and development, green involvement), characterized as the portion of HRM that engages with environmental sustainability standards [25]. Green HRM has been considered a key factor in improving green OB, including recruiting and selection, training and development, performance evaluation, and remuneration, similar to traditional HRM [26]. While the difference is that green HRM is more focused on being concerned about environmental issues, including green hiring (hiring employees with specific environmental competencies and with general sensitivity toward the environment), green training, and involvement (developing environmental competencies and skills and engaging employees in green behaviours), enhancing employee engagement and lower expenses. Furthermore, green HRM in organisations, such as carpooling, virtual training, job sharing, teleconferencing, online interviewing, and recycling, assist organisations in reducing their employees’ carbon footprint [27].

Green OB has defined as an employee’s voluntary behaviours that contribute to the organisation’s environmental goals [28]. organisations with higher green OB are willing to hire employees with higher environmental capabilities and a desire to participate in environmental-related activities [29]. Employees’ green behaviour may improve due to a good green HRM policy [30]. In particular, green training offers employees green knowledge and skills, improving their green capacities to anticipate environmental concerns [31]. Employees become more aware of environmental norms, act more proactively, and support the spread of environmental ideals to inspire employees to engage in voluntary green behaviour. Several studies demonstrate that green HRM activities are required to promote organisational citizenship behaviour at work [32–34]. Thus, we advance the following hypothesis:

H2: Green HRM will positively effect green OB.

### 1.3 Environmental management accounting and green organisational behaviour

Given the deterioration of the ecological environment in recent years, the environmental development of society is facing enormous challenges [35]. Guided by the rational development of resources and focusing on environment development as a central element, green OB encompasses an individual’s discretionary behaviours directed toward a green environment but not required by the organisation. Bringing EMA closer to green OB could be a welcome move to reconcile organisations [36].

We enumerated several roles for EMA in the organisation. However, this literature has rarely crossed and less fully understands EMA’s potential contribution to green OB. EMA approaches contribute to the support of organisational practices, which benefits both the organisation’s environmental behaviour and the achievement of environmental accounting goals. We need holistic thinking about how green OB is implemented. EMA is about figuring out how to put intangible and tangible environmental aspects into monetary (MEMA) and physical (PEMA) attributes that management utilizes to make short- and long-term decisions. When enterprises implement EMA and follow suit, organisations will also have such thoughts and application ideas of strategic management accounting to realize green OB.

Schaltegger, Viere [37] used a change management method to identify barriers to EMA implementation in British and German organisations; they argued that most organisations are in the reactivating stage of EMA change. Gunarathne and Lee [38] proposed an empirical example of EMA practice at a Sri Lankan hotel, demonstrating how a hotel successfully transitions from survival to environmental integration.

This study argues that EMA has a driving effect on the hotel industry green OB based on the analysis above. MEMA is focused on intangible environmental aspects of green OB, and PEMA is focused on tangible environmental aspects of green OB. EMA information provides important information for enterprise decision-making associated with green OB. Thus, we advance the following hypothesis:

H3a: MEMA will positively effect green OB.

H3b: PEMA will positively effect green OB.

### 1.4 The mediation role of green HRM

Environmental issues have become more concerned with China’s prevailing environmental incentive mechanisms, such as carbon and emission trading [39]. The purpose of EMA is to measure the daily cost of enterprises to reduce and weaken the pollution or damage to the environment caused by their production and operation activities [40]. Environmental organisations are increasingly willing to disclose information through EMA. More organisations with better EMA are willing to actively disclose environmental information to investors and stakeholders and send good signals to the outside world to distinguish themselves from poor ones [41].

That is why more managers are becoming interested in EMA information and utilising EMA tools to assist in the organisation management process. Green HRM guides employees to align their behaviours with the organisation’s environmental accounting objectives, which is a supporting role by EMA. It is an effective way to signal to employees the organisation’s commitment to sustainability [42].

Green HRM emphasizes integrating the concept of pursuing environmental protection and sustainability into the specific human resource management measures in organisation and management. First, in the recruitment process, the enterprise conveys the organisation’s preference for environmental protection by publicizing the company’s environmental protection concept [26]. This concept can attract candidates with high environmental awareness. At the same time, it emphasizes the performance of environmental protection responsibilities in the interview process, making it easier to screen candidates consistent with the organisation’s environmental protection values. Such measures enable successful employees to feel the organisation’s attention to environmental protection before officially entering the organisation, which helps to enhance employees’ green awareness. Second, by popularizing environmental protection knowledge and providing environmental protection skill training, the company can promote environmental protection knowledge and mutual assistance among employees. The company can also make employees aware of organizing environmental protection activities and enhancing their motivation to engage in green behaviour [27]. Finally, the organisation implements green HRM to convey its support for green behaviour. Doing so encourages employees to participate in the enterprise’s environmental protection practices and achieve the organisation’s green development goals [30].

Paying attention to environment-oriented HRM practices helps to improve organisational citizenship behaviour in the workplace [43]. A good green HRM policy may lead to employees’ green behaviour [30]. Green training, in particular, offers employees with green knowledge and skills, boosting their green capacities to recognize and mitigate environmental problems. As a result, employees become more aware of environmental norms, act more proactively, and support the spread of environmental ideals to inspire employees to engage in voluntary green behaviour.

Incorporating EMA with green HRM provides clear information to green OB concerning their role in the green OB. It increases EMA adoption among intrinsically motivated green OB. Thus, we advance the following hypothesis:

H4a: Green HRM will mediate the relationship between MEMA and green OB.
H4b: Green HRM will mediate the relationship between PEMA and green OB.

**Fig 1:**
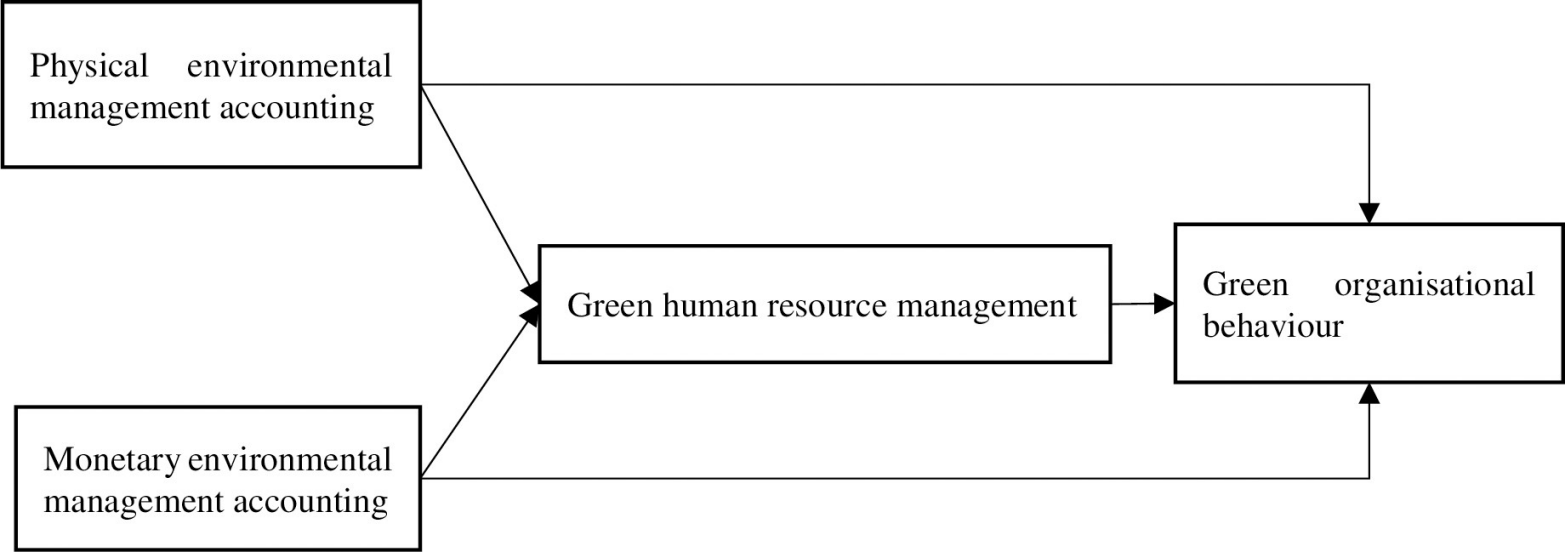
Research model.

Fig 1 showed the research model developed for this purpose.

## 2. Methodology

### 2.1 Measures

This study variable was measured using well-established scales. A five-point Likert scale ranging from 1 (“strongly disagree”) to 5 (“strongly agree”) was used throughout.

#### Environmental management accounting (EMA)

We adopted the Environmental management accounting scale compiled by Ferreira, Moulang [13], containing 13 questions from 2 dimensions (MEMA and PEMA). A sample item of MEMA is “Classification of environment-related costs.” Its Cronbach’s alpha was 0.898 in this study. A sample item of PEMA is “Product inventory analyses (i.e., the specification of the types and quantities of materials and energy required and the number of residues released to the environment).” Its Cronbach’s alpha was 0.861 in this study.

#### Green human resource management (GHRM)

We adopted the Green Human Resource Management scale by Mousa and Othman [44], containing three dimensions and eight items. A sample item is “In addition to other criteria, employees are selected based on environmental standards.” Its Cronbach’s alpha was 0.899 in this study.

#### Green organisational behaviour (OB)

We adopted Graves, Sarkis [45] green organisational behaviour scale, including 10 items. A sample item is “At work, I offer ideas for reducing our impact on the environment.” It bs Cronbach’s alpha was 0.918 in this study.

### 2.2 Sample and procedure

This quantitative research study measures EMA’s and green HRM’s effects on green OB in the hotel industry. We used a multi-source survey to test our hypotheses, one for HR managers and one for accounting managers. This method enables us to avoid biases due to single respondents. In this way, we can triangulate information (as they are asked to two respondents) related to environmental management accounting and green human resources, accounting managers who were deemed appropriate participants given their involvement in the day-to-day financial and operational activities of the enterprise, in addition to their likely involvement in activities related to the environment, green HRM, and green OB [46]. In contrast, the information related to green HRM comes from HR managers, the most knowledgeable respondent in human resource management.

This study chose participants (HR managers and accounting managers) in China’s hotel industry. The study was conducted from May to October 2021. According to the 2021 China Hotel Industry Development Report, China’s hotel industry has 279,000 hotels. We identified contact information for hotel management personnel through the hotel management personnel training conference. The hotel management personnel answered the questionnaire’s basic information and were asked to forward the questionnaire to the accounting manager and human resource management manager for completion. We explained the principles of the questionnaire to HR managers and accounting managers. We requested that they complete the questionnaire by e-mail, thereby ensuring the basic conditions of structural equation model analysis.

We use convenience sampling. The survey was sent 600 questionnaires and received 383 questionnaires, confirming the requirement that the ratio of the number of questions of the questionnaire to sample size is 1:10 [47], and the response rate was 63.8%. The reason for the low recovery rate is that some company policies restricted the surveys, and some respondents considered environmental issues a sensitive matter. Our research design allowed us to obtain measures of predictor and criterion variables from different sources, which helped control method bias [48].

Furthermore, green HRM and green OB efforts help the hotel industry stand out from competitors in a sector where fierce competition is common. These hotels have emphasized environmental obligations, implementing corporate social responsibility strategies that prioritize environmental conservation [49]. Environmental training for both staff and managers, such as reducing, recycling, and replacing waste and utilizing electricity and water efficiently, is an important ecological practice at these hotels. This training denotes a focus on environmental standards to create a green and protracted hotel industry to appeal to target consumers.

## 3. Results

### 3.1 Respondent characteristics analysis

The frequency distribution of individual basic data is summarized as follows: In terms of gender, 245 males (64%) and 138 females (36%); The largest number of respondents who chose age was 36 to 40 years, with 159 (41.5%), followed by 31 to 35, with 88 (22.9%).The largest number of respondents who chose education level was university-undergraduate-level, with 176 (46.0%), followed by university-graduate-level, with 146 (38.1%). The largest number of respondents who chose monthly salary was CNY 15001–20000, with 186 (48.6%).The largest number of respondents who chose firm tenure was 11–15 years, with 225 (58.7%).

### 3.2 Common method bias

Various methods were used to test the CMB effect. First, Herman’s one-factor test. Factor analysis through SPSS was performed. The results showed that more than one factor was extracted, and the maximum factor explanation degree was 35.462% (<50%) [50].

### 3.3 Confirmatory factor analysis

This study used different fit indices were examined to evaluate the path model’s fit. The chi-square significative degree relates to the sample sizes [51], so Wheaton, Muthen [52] propose that relative chi-square (CMIN/df). According to Schumacker and Lomax (2004), χ2/df ratio < 3.00 is satisfactory, the IFI, CFI, and TLI must be greater than 0.90 and the RMSEA must be less than 0.05 [53]. This study evaluated four models with the composite index of overall fitness, the result of which indicates that the four-factor measurement model was superior to the other three models. The results show that CMIN = 943.201, DF = 428, CMIN/DF = 2.204, *p* < 0.001(i.e., CMIN/DF < 3). The results demonstrate CFI = 0.856, TLI = 0.913, RMSEA = 0.056 to indicate an acceptable fit [54], as shown in Table 1.

**Table 1:** Model fit from measurement model comparison.

Models	CMIN	Df	CMIN / Df	IFI	TLI	CFI	RMSEA
4 Factor (Measurement model)	943.201	428	2.204	0.920	0.913	0.920	0.056
3 Factor Model ^a^	1542.886	431	3.577	0.828	0.813	0.827	0.082
2 Factor Model ^b^	2335.872	433	5.395	0.705	0.681	0.703	0.107
1 Factor Model ^c^	3087.632	434	7.114	0.588	0.556	0.586	0.127

Note(s): N = 383, All models were compared with the four-factor model (measurement model)

Model a: Monetary environmental management accounting and physical environmental management accounting combined into one factor; Green human resource management combined into one factor; organisational green behaviour into another factor

Model b: Monetary environmental management accounting, Physical environmental management accounting and green human resource management combined into one factor; organisational green behaviour into another factor

Model c: All constructs combined into one factor

χ2 = chi-square; Df = Degrees of Freedom; IFI = Incremental Fit Index; TLI = Tucker–Lewis Index

CFI = Comparative Fit Index; RMSEA = Root Mean Square Error of Approximation

### 3.4 Convergent validity

The construct validity of the measuring instruments was assessed through their convergent and discriminant validity. All the measurement scales in this study inquiry satisfy Fornell and Larcker [55] requirements. All constructs in the study have high convergent validity as the individual item loaded on their respective construct in the range of ≥0.662 to 0.844. The AVE was ≥ 0.525 to 0.561. Thus, all the measuring instruments had convergent and discriminant validity [55]. Convergent validity shown in Table 2.

**Table 2:** Convergent validity.

Variables	Items	Factor Loading	CR	AVE
Monetary environmental management accounting	Identification of environment-related costs	0.760	0.898	0.525
	Estimation of environment-related contingent liabilities (e.g. EPA fines)	0.715		
	Classification of environment-related costs	0.711		
	Allocation of environment-related costs to production processes	0.743		
	Allocation of environment-related costs to products	0.710		
	Introduction or improvement to environment-related cost management	0.738		
	Creation and use of environment-related cost accounts	0.731		
	Development and use of environment-related key performance indicators (KPIs)	0.687		
Physical environmental management accounting	Product life cycle cost assessments	0.844	0.864	0.561
	Product inventory analyses (i.e. the specification of the types and quantities of materials and energy required and the amount of residues released to the environment)	0.748		
	Product impact analyses (i.e. assessment of the environmental effect of competing product designs)	0.774		
	Product improvement analysis (i.e. identification of opportunities for reduction of environmental impact)	0.705		
	Product life cycle cost assessments	0.662		
Green human resource management	Environmentally conscious candidates are more valued by hotels	0.725	0.899	0.528
	Hotels can attract employees through environmental promises	0.702		
	The hotel can carry out environmental protection training for employees, such as providing practical activities for employees to participate in environmental management	0.713		
	The hotel where I work provides environmental training for management	0.726		
	Environmental responsibilities are included in the hotel job description	0.728		
	The hotel values staff’s participation in environmental issues	0.763		
	Hotels select employees based on environmental criteria when hiring	0.752		
	One of hoteliers’ goals is green performance management and compensation	0.699		
Green organisational behaviour	I try to learn more about the environment	0.724	0.919	0.530
	I offer ideas for reducing our impact on the environment	0.737		
	I share my knowledge about the environment with others	0.753		
	I apply new ideas for reducing our impact on the environment	0.731		
	I help create green processes and products	0.735		
	I perform environmental tasks that are not required by the hotel	0.712		
	I question practices that are likely to hurt the environment	0.713		
	I recycle and reuse materials, such as double-sided printing	0.713		
	I try to reduce energy use, such as turning off lights	0.748		
	I join in environmental activities that are not required by my job	0.713		

### 3.5 Correlation and discriminant validity

The effect size of Pearson r correlation varies between -1 (a perfect negative correlation) to +1 (a perfect positive correlation), the value of r varies around 0.1 showing the effect size is low and r varies more than 0.5 showing the effect size is large [56]. As shown in Table 3, correlation analysis was calculated by SPSS adopted the Pearson correlation coefficient based on the average number of items. There is a significant positive correlation between every two variables. In addition, discriminant validity adopts the mode between latent variable correlation and latent variable pair correlation confidence interval detection methods [57]. This study adopts the standard correlation coefficient ±1.96 standard deviation through AMOS. None of the confidence interval values in all the brackets have a value covering 1.00. The results show that the correlation between potential variables is distinguishable and has discriminant validity.

**Table 3:** Correlation and discriminant validity.

	Mean	Std.Dev	MEMA	PEMA	GHRM	GOB
MEMA	3.939	0.538	1			
PEMA	3.486	0.697	0.419** (0.432, 0.506)	1		
GHRM	3.672	0.565	0.398** (0.401, 0.479)	0.461** (0.456, 0.546)	1	
GOB	3.731	0.587	0.516** (0.524, 0.606)	0.449** (0.444, 0.530)	0.443** (0.442, 0.532)	1

Note: () is standard correlation coefficient ±1.96 standard deviation through AMOS.

* *p* ≤ 0.05.

** *p* ≤ 0.01

### 3.6 Testing hypotheses

In this study, we examined our hypotheses with a structural equation model (SEM), using Amos software. As shown in Table 4, MEMA positively effects green HRM (*β* = 0.262, *p* < 0.01), thereby supporting Hypothesis 1a. PEMA positively effects green HRM (*β* = 0.378, *p* < 0.01), thereby supporting Hypothesis 1b. Green HRM positively effects green OB (*β* = 0.223, *p* < 0.01), supporting Hypothesis 2. MEMA positively effects on green OB (*β* = 0.373, p < 0.01), supporting Hypothesis3a. PEMA positively effects on green OB (*β* = 0.200, *p* < 0.01), supporting Hypothesis 3b.

**Table 4:** Direct effect and indirect effect.

	Path	Estimate	95% Confidence Interval		
			BC/PC *p*-value	BC	PC
				Lower-upper	Lower-upper
Direct effects	MEMA →GOB	0.373	0.001/0.001	0.266–0.476	0.266–0.476
	PEMA →GOB	0.200	0.001/0.002	0.077–0.328	0.072–0.323
	MEMA →GHRM	0.262	0.001/0.001	0.124–0.399	0.109–0.389
	PEMA →GHRM	0.378	0.001/0.001	0.250–0.507	0.243–0.502
	GHRM →GOB	0.223	0.001/0.001	0.102–0.332	0.104–0.333
Indirect effects	MEMA→GHRM→GOB	0.059	0.001/0.002	0.020–0.114	0.017–0.109
	PEMA→GHRM→GOB	0.084	0.001/0.001	0.040–0.146	0.036–0.140
Total effects	MEMA →GOB	0.431	0.001/0.001	0.333–0.530	0.334–0.530
	PEMA →GOB	0.284	0.001/0.001	0.171–0.379	0.164–0.391

Notes: MEMA = Monetary environmental management accounting; PEMA = Physical environmental management accounting; GHRM = Green human resource management; GOB = Green organisational behaviour.

Hypothesis 4 proposed that green HRM mediates the relationship between MEMA/ PEMA and green OB. Further analyses were performed to confirm the magnitude and statistical significance of the indirect effects of bootstrapping in 2000, with a 95% confidence interval [58]. The result shows that the indirect effects of MEMA on green OB via green HRM were significant within 95% confidence intervals (*β* = 0.059, *p* < 0.01), Thus, Hypothesis H4a is supported. PEMA on green OB via green HRM was significant within 95% confidence intervals (*β* = 0.084, *p* < 0.01), Thus, Hypothesis H4b is supported.

To further test the hypothesized results, we performed a second test of the hypothesis using hierarchical linear regression via SPSS 25 software. As shown in Table 5, the maximum variance inflation factor (VIF) coefficient of all models was 1.277, which was less than the threshold of 5 [59, 60], so no serious multicollinearity was found. Moreover, all models except Model 1 and Model 4 reached a significant level of F values (*p*<0.001), indicating that the models were statistically significant.

**Table 5:** Hierarchical linear regression analysis.

Dependent variable Independent variable	Green HRM			Green OB					
	Model 1	Model 2	Model 3	Model 4	Model 5	Model 6	Model 7	Model 8	Model 9
(constant)	***	***	***	***	***	***	***	***	***
Gender	-0.047	-0.046	-0.024	0.004	0.025	0.005	0.026	0.018	0.034
Age	0.013	0.019	0.000	-0.007	-0.012	0.002	-0.019	-0.004	-0.019
Education level	-0.022	0.021	-0.006	-0.113*	-0.103*	-0.059	-0.098	-0.065	-0.096
Monthly salary	0.007	-0.013	0.003	0.034	0.031	0.008	0.030	0.012	0.029
Firm tenure	0.096	0.063	0.067	0.083	0.041	0.040	0.054	0.023	0.035
MEMA		0.397***				0.505***		0.393***	
PEMA			0.456***				0.444***		0.309***
Green HRM					0.437***			0.283***	0.296***
Adj R^2^	-.002	.151	.204	.009	.198	.260	.205	.326	0.273
F value	.827	12.345***	12.345***	1.730	16.763***	23.364***	17.419***	27.416***	21.461***
D-W	/	1.560	1.652	/	1.770	1.903	1.568	2.024	1.737
Max VIF	1.042	1.042	1.045	1.042	1.045	1.042	1.042	1.211	1.277

Note

*p<0.01

**p<0.05

***p<0.001.

In Model 2, MEMA had a positive effect on green HRM (*β* = 0.397, *p* < 0.001). Hence, H1a was supported. In Model 3, PEMA had a positive effect on green HRM (*β* = 0.456, *p* < 0.001). Hence, H1b was supported. In Model 5, green HRM had a positive effect on green OB (*β* = 0.437, *p* < 0.001). Hence, H2 was supported. In Model 6, MEMA had a positive effect on green OB (*β* = 0.505, *p* < 0.001). Hence, H3a was supported. In Model 7, PEMA had a positive effect on green OB (*β* = 0.444, *p* < 0.001). Hence, H3b was supported.

According to Baron and Kenny [61], we utilized the three-step regression method to test whether green HRM has a mediating effect. Step 1: MEMA (Model 6)/PEMA (Model 7) has a direct effect on green OB. Step 2: MEMA (Model 2) /PEMA (Model 3) has a direct effect on green HRM. Step 3: After green HRM is introduced, the effect of MEMA/PEMA on green OB still exists. As shown in Model 8/ Model 9, *p* < 0.001 with statistical significance. With MEMA/PEMA, the *β* coefficient dropped to 0.393/0.309, indicating that green HRM plays a positive partial mediates effect relationship between MEMA/PEMA and green OB. To sum up, H4 was supported.

## 4. Discussion

### 4.1 Theoretical implications

This research has some contributions. The conceptual model presented in this study can inform the hotel industry about EMA and green HRM effects on green OB. The data below is from AMOS software path analysis results.

First, EMA (MEMA, Beta = 0.373, p < 0.01; PEMA, Beta = 0.200, *p* < 0.01) and green HRM (Beta = 0.223, *p* < 0.01) positively effects green OB. The hotel industry is one of the main contributors to the economy in China. However, it is also the highest contributor to environmental issues. Previous research has shown that EMA has a significant positive effect on environmental performance [11, 12], which is consistent with the results of this study. Imbued with the appropriate knowledge and skills, MEMA and PEMA implement a green enterprise policy, which is more likely to affect green OB. Adopting green HRM includes cost reduction, talent attraction, and retention [42]. The human resource manager formulates the human resource training program, and EMA provides the important information that formulates the program. Both have a significant effect on green OB. Green HRM improves employees’ environmental awareness and skills through green-oriented recruitment, training, performance management, assessment, compensation, and welfare, motivating them to engage in green behaviours through rewards and benefits [62]. The above results are consistent with previous studies [63, 64].

Second, EMA (MEMA, Beta = 0.262, *p* < 0.01; PEMA, Beta = 0.378, *p* < 0.01) positively effects green HRM. Previous research has shown that green human capital, green structural capital, and green relational capital are positively associated with environmental management accounting [8]. It is consistent with the results of this study. Imbued with the appropriate knowledge and skills, MEMA and PEMA implement green enterprise policy, which is more likely to affect green HRM. Aggressively pursuing the adoption of environmentally friendly activities, such as green HRM, is becoming crucial to mitigate environmental problems [65].

Third, green HRM mediates the relationship between EMA and green OB, that is, green HRM mediates the relationship between MEMA and green OB (Beta = 0.059, *p* < 0.01), and the relationship between PEMA and green OB (Beta = 0.084, *p* < 0.01). The purpose of EMA is to measure the daily cost of enterprises to reduce and weaken the pollution or damage to the environment caused by their production and operation activities [40]. Green HRM guides employees to align their behaviours with the organisation’s environmental accounting objectives, which is a supporting role by EMA. It is an effective way to signal to employees the organisations’ commitment to sustainability [42]. Paying attention to environment-oriented HRM practices helps to improve organisational citizenship behaviour in the workplace [43].

### 4.2 Practical significance

From a practical perspective, this study offers numerous key implications, especially for business professionals and policymakers, on leveraging environment management by accounting for its monetary and physical dimensions, beating rivals in the markets, and attaining enterprises’ environmental management goals.

First, this study contributes to advancing understanding and explains what causes EMA and organisation green behaviour. Based on this study’s results, we suggest that EMA is a strategic resource enterprise should leverage to shape and implement green HRM, influencing green organisational behaviour. While applying to the green HRM link, we suggest that green HRM is a critical resource as any other organisational resource that should be valued. It becomes difficult for the competing enterprises to imitate, employees feel the humanistic care of the enterprise and the cultivation of various green measures, green training, and involvement. Similarly, this study confirmed that enterprises should design and implement green HRM practices to attract, train, motivate, and retain green employees to enhance the green OB.

Second, we suggest that EMA is beneficial to enterprises to have a good image to stakeholders. The latter has become more demanding and pressurizing enterprises to go green in all its monetary and physical processes, products, and services. Our study suggests that enterprises should emphasize and reinforce the necessary EMA. EMA is essential for acquiring, developing, and sustaining the management accounting processes, which help support enterprises’ strategies to compete with competitors. Therefore, we suggest that enterprises invest in EMA; what is important is that EMA should not be a knee-jerk reaction to stakeholder pressure but proactive measures to reduce negative environmental impact.

Third, given that green HRM has gained attention in recent years, using this model of green HRM in hotel industries in China could help maximize the organisation’s green behaviour and use green HRM as a strategy to sustain competitiveness. This study shows that green HRM has a mediating role in the effect of EMA on green OB. Human capital is rooted in employees and can disappear when employees leave. Environmental accounting knowledge is embedded in employees’ behaviours. Therefore, companies need to develop green management, attract the best human capital, and develop and cultivate their existing employees to contribute to developing the green OB.

## 5. Conclusions, limitations and future research

This study explored the relationship between EMA and green OB by presenting a novel research framework. First, this study reveals the effect of two dimensions of EMA (MEMA and PEMA) on green OB, providing new empirical evidence for environmental management research. Second, this study tests the mediator role of green HRM in the relationship between EMA and green OB as a unique contribution to the existing green OB literature.

This research has some limitations. First, this study explores the effect of outcome variables at the level of organisation. Future research can consider the impact of green HRM on green technology innovation [66]. Previous research has found that guanxi [67, 68] and psychological contracts [68] influence employee green behaviour. Follow-up research can also start from these aspects and conduct cross-level research to explore its impact on individual behaviour to promote the development of this field.

Second, this study focuses on the mediating role of green HRM without considering other variables. Other potential mediating variables can be explored later. Considering the organisational characteristics important in the workplace, future research can explore other characteristics. For example, research might investigate workplace climate and employee moral cognition in combination with other leadership styles (e.g., green transformational leadership) on the implementation effects of green OB.

Third, this study has some limitations in sample size and scope regarding research data. This study constructed the research model’s EMA, green HRM, and green OB. However, due to time and resource constraints, only for China hotel accounting manager and HR manager collected the survey data, which to a certain extent affected the external validity of the results. Employees from different industries were not involved, and samples of employees from other countries’ companies were not added to compare cultural differences with employees from China companies. Future research can expand the number of cities, industries, and enterprises studied to improve the representativeness and universality of samples and the generalization of any conclusions.

Final, this study only selects the personal characteristics of corporate leaders as control variables, not the corporate characteristics. Follow-up studies could investigate enterprise characteristics as control variables.

## Supporting information

S1 Data (XLSX)
